# Development, circuitry, and function of the zebrafish cerebellum

**DOI:** 10.1007/s00018-023-04879-5

**Published:** 2023-07-25

**Authors:** Sol Pose-Méndez, Paul Schramm, Komali Valishetti, Reinhard W. Köster

**Affiliations:** Cellular and Molecular Neurobiology, Zoological Institute, Technische Universität Braunschweig, 38106 Braunschweig, Germany

**Keywords:** Zebrafish, Cerebellum, Purkinje cell, Granule cell, Evolution, Cerebellar circuitry, Cerebellar development

## Abstract

The cerebellum represents a brain compartment that first appeared in gnathostomes (jawed vertebrates). Besides the addition of cell numbers, its development, cytoarchitecture, circuitry, physiology, and function have been highly conserved throughout avian and mammalian species. While cerebellar research in avian and mammals is extensive, systematic investigations on this brain compartment in zebrafish as a teleostian model organism started only about two decades ago, but has provided considerable insight into cerebellar development, physiology, and function since then. Zebrafish are genetically tractable with nearly transparent small-sized embryos, in which cerebellar development occurs within a few days. Therefore, genetic investigations accompanied with non-invasive high-resolution in vivo time-lapse imaging represents a powerful combination for interrogating the behavior and function of cerebellar cells in their complex native environment.

## Introduction

**Fig. 1:**
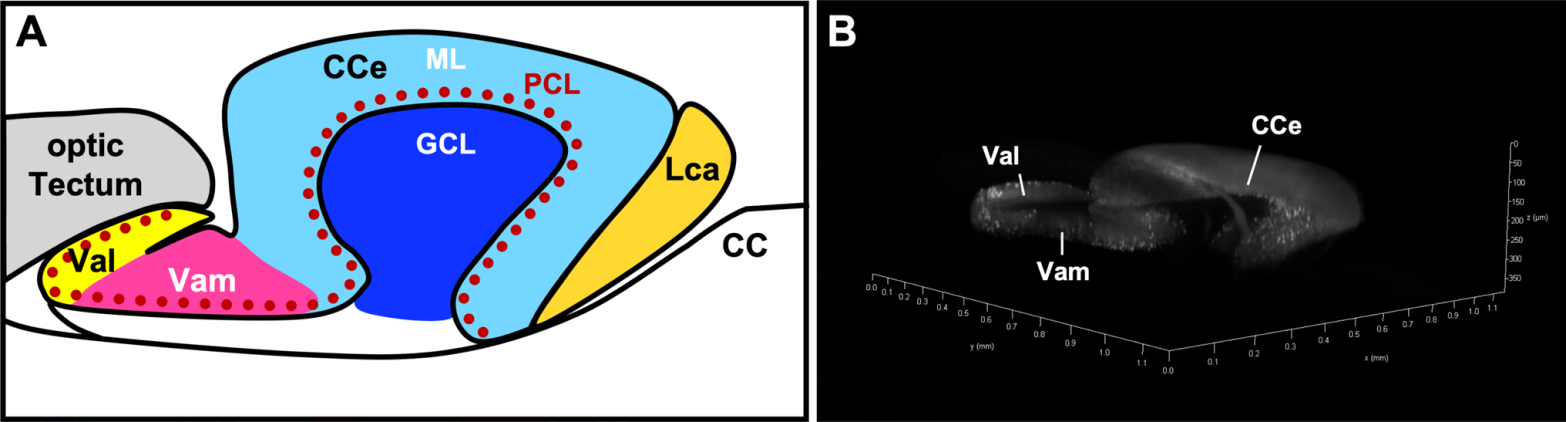
The adult zebrafish cerebellum. **a** Schematic drawing of a sagittal view of the adult zebrafish cerebellum, the individual cerebellar compartments are differently colored. (**b** provided by Sven Wargenau) Three-dimensional reconstruction of the cerebellum from a cleared brain of an adult Tg(-7.5ca8:EGFP)^bz12^ zebrafish with green fluorescence in the Purkinje cell population. Note the wing-shaped structures of the valvular lateralis. *CC* crista cerebellaris, *CCe* corpus cerebelli, *GCL* granule cell layer, *LCa* lobus caudalis, *ML* molecular layer, *PCL* Purkinje cell layer, *Val* valvula lateralis, *Vam* valvula medialis. Anatomical terminology according to [3]

The cerebellum—meaning the small brain in Latin language—forms an own and anatomically easily distinguishable compartment of the brain in jawed vertebrates (gnathostomes). Although small in size, it contains about half of the neurons of the entire brain, and these neurons are organized in a paracrystalline manner found in three separated layers [1]. These layers can be viewed in a simplified manner as input layer [innermost layer (granule cell layer) formed by cerebellar interneurons], processing layer [outermost layer (molecular layer) consisting mostly of neuropil and harboring the vast majority of cerebellar synaptic connections], and the output layer [intermediate layer (Purkinje cell layer) containing projection neurons] (Fig. 1a). While this cellular organization can be found from teleosts to mammals, there are some obvious gross anatomical differences in the appearance of the cerebellum. In teleost fishes, the cerebellum forms a bell-shaped structure with a smooth surface in the antero-dorsal hindbrain. In birds and mammals instead, the cerebellum is highly folded in a stereotypical manner allowing to assign numbered lobes to these folds. Therefore, on sagittal sections, the avian and mammalian cerebellum reminds of a cauliflower, and from medial to lateral the vermis, paravermis, and hemispheres can be distinguished as further subcompartments that are not distinguishable anatomically in bony fish. With respect to the cytoarchitecture, Purkinje neurons of the avian and mammalian cerebellum extend axons as projection neurons to the base of the cerebellum, where their efferent structures, four different neuronal nuclei termed the deep cerebellar nuclei are localized. The equivalents of these deep cerebellar neurons do not form condensed neuronal nuclei in teleost but reside in close neighborhood to their afferents, the Purkinje cells; therefore, anatomically deep cerebellar nuclei neurons in teleosts cannot be identified [2, 3]. Nevertheless, the different neuronal cell types, their connectivity, and function have been highly conserved from teleosts to humans [4] making the cerebellum one of the highest evolutionary conserved compartments throughout jawed vertebrate brains. Therefore, studying teleostian cerebellar development, physiology, and function is a rewarding research field not only for teleost basic neuroscience research, but also for providing fruitful information to better understand the human cerebellum. This review aims to summarize the current knowledge about the development, neuronal differentiation, and physiology of cerebellum research in the teleost zebrafish to attract further generations of neuroscientists to this fascinating brain structure with intricate functions ranging from locomotor control and motor learning to regulating socio-emotional behavior.

## Early developmental steps of zebrafish cerebellum formation

The differentiated zebrafish cerebellum can be subdivided into three compartments (Fig. 1) [3]. The corpus cerebelli (CCe) is the most prominent part and is evolutionary related to the spinocerebellum or vermis in mammals involved in motor coordination and socio-emotional regulation. In the anterior direction, the corpus extends into the valvula cerebelli [subdivided into a medial (Vam) and lateral (Val) part] that is specific to ray-finned fish with a yet unclear function. Posterior to the corpus cerebelli, the lobus caudalis (LCa) together with the laterally positioned eminentia granularis are called the caudolateral lobe, which represents the vestibulocerebellum responsible for body posture and balance control, the evolutionarily oldest part of the vertebrate cerebellum.

### Early patterning processes

**Fig. 2:**
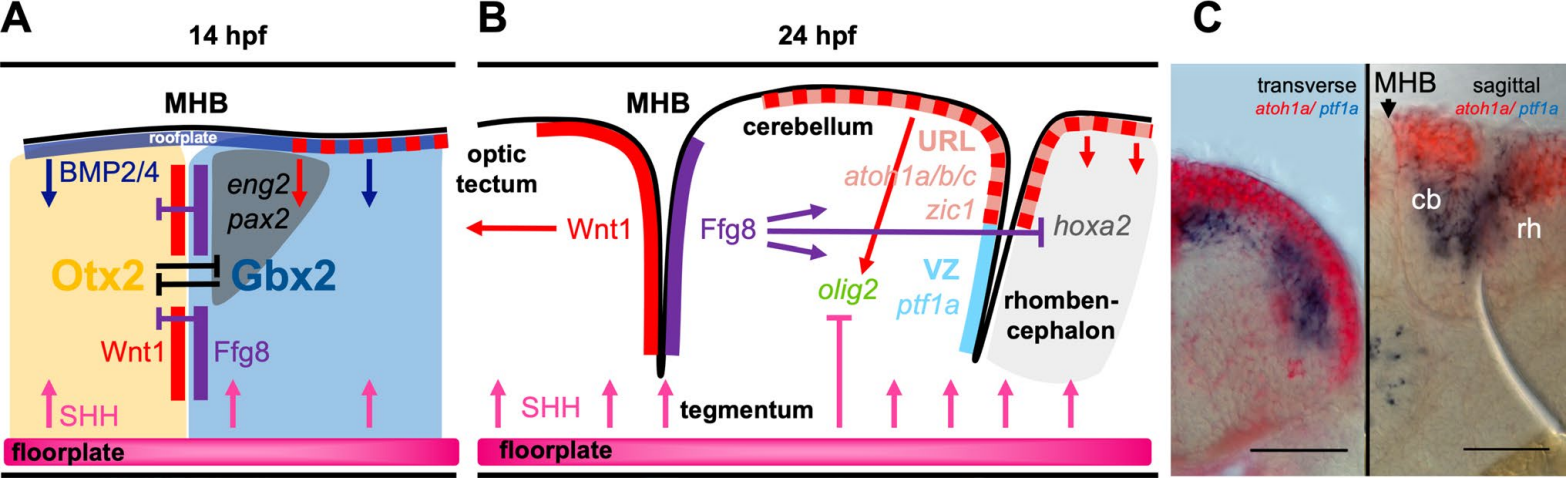
Molecular processes leading to the establishment of a patterned cerebellar primordium in zebrafish. **a** Schematic drawing of the establishment of the midbrain hindbrain boundary (MHB), a key secondary organizer tissue involved in inducing the cerebellar primordium in the dorso-anterior hindbrain. These processes are already initiated during mid-gastrulation stages, but gene expression is maintained in 14hpf embryos, where it can be assigned well to embryonic neuroanatomical structures. **b** At 24hpf, the cerebellar primordium is well established and patterned including the two major germinal zones, the upper rhombic lip (URL) and the ventricular zone (VZ) that give rise to all neurons in the adult cerebellum. (**c** Provided by Andreas Babaryka) Overlay of transmitted light images with results from double in situ hybridization at 36hpf against the mRNA of *atoh1a* (red) and *ptf1a* (blue) as molecular markers for these two juxtaposed proliferation zones. Transverse (left) and sagittal (right) vibratome sections through the developing cerebellum are displayed, scale bar. 50 µm. *cb* cerebellum, *MHB* midbrain-hindbrain boundary, *rh* rhombencephalon

The cerebellum is located in the anterior-most hindbrain and originates from the dorsal part of rhombomere 1 [1]. Early cerebellar development, therefore, occurs under the influence of the midbrain hindbrain boundary (MHB), or isthmus, a dorso-ventrally oriented boundary tissue with secondary organizer function in the brain [5]. Like in mice, this organizer in zebrafish forms at the border where expression of the two transcription factors *orthodenticle 2* (*otx2*) and *gastrulation brain homeobox 1* and *2* (*gbx1*, *gbx2*) is juxtaposed mediated by *pou domain homeobox gene 2* (*pou2*) [6–8]. The organizer itself is demarcated by an anterior stripe of *wnt family member 1* (*wnt1*) and a posterior half of *fibroblast growth factor 8* (*fgf8*) expression (Fig. 2a). These diffusible growth factors trigger further downstream the expression of genes involved in maintenance of isthmic tissue and in further patterning of the cerebellar primordium including *fibroblast growth factor 17 (fgf17)*, *ezrin/radixin/moesin* proteins* (erm)*, *polyoma enhancer activator 3* (*pea3*) or *sprouty4* (*spry4*), and members of the *engrailed* (*eng*) transcription factor family among others. Among the signal transduction molecules, the role of FGF8 in zebrafish is well understood. Lack of its expression in the *acerebellar* mutant leads to the absence of a cerebellar primordium [9]. This phenotype can be recapitulated by pharmacological inhibition of FGF-signal transduction during onset of gastrulation (50% epiboly) with the FGF-receptor (FGFR) inhibitor SU5402, yet if FGFR inhibition is initiated slightly later at mid-gastrulation stages (70% epiboly), the cerebellum forms properly in nearly all treated embryos [10]. Thus, induction of the cerebellar primordium occurs during early gastrulation stages in zebrafish. Here, FGF8 expression at the MHB serves to suppress expression of the *homeobox protein a2* gene (*hoxa2*) in the anterior hindbrain and to delimit the expression of *otx2* to the midbrain [11]. This suggests that FGF8 spatially defines rhombomere 1 in the hindbrain as the only compartment without *hox* gene expression, enabling its development into cerebellar tissue from the dorsal half. As a dorsal neural tube structure, patterning of the cerebellar anlage occurs in parallel from the roof plate involving signaling via bone morphogenetic proteins (BMPs) and the expression of transcription factors of the *paired homeobox gene 2, 5* and *8* (*pax2,5,8*) family. For example, injection of a function-inhibiting antibody against Pax2 impairs the maintenance of its own expression and compromises the expression of cerebellar primordium key genes *wnt1* and *engrailed2* resulting in failure of the embryo to establish a proper cerebellar primordium [12].

**Fig. 3:**
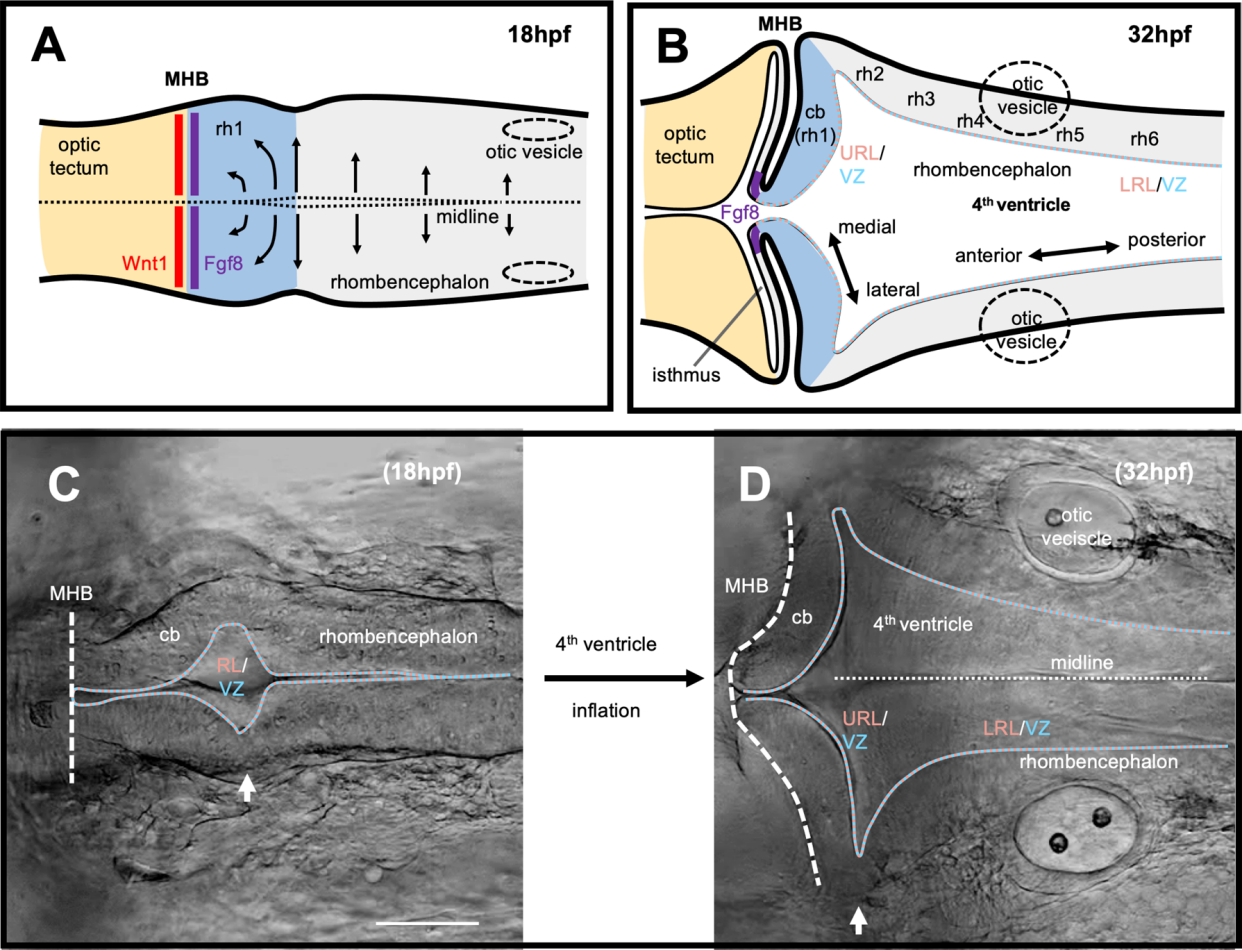
Morphogenetic processes leading to the bilaterally wing-shaped cerebellar primordium in zebrafish. Dorsal views are shown in schematic drawings (**a**, **b**) and corresponding images recorded by transmitted light stereomicroscopy (**c**, **d**, provided by Martin Distel). *cb* cerebellum, *LRL* lower rhombic lip, *MHB* midbrain–hindbrain boundary, *rh* rhombomere, *URL* upper rhombic lip, *VZ* ventricular zone. Scale bar: 100 µm

In parallel to these molecular processes, morphogenetic changes occur by the inflation of the 4th ventricle during mid-somitogenesis stages (18 h postfertilization, hpf, Fig. 3a, c), resulting in the lateral displacement of the walls of the hindbrain neural tube [13]. Because cells at the MHB continue to adhere along the dorsal midline, the two halves of rhombomere 1 rotate by almost 90 degrees forming characteristic bilateral wing-shaped structures from 20hpf onwards (Fig. 3b, d, 32hpf) allowing to visually distinguish the cerebellar primordium for the first time [14]. Due to these morphogenetic rearrangements, the ventricular neurogenic zone usually facing the midline of the hindbrain neural tube is displaced in the cerebellum and delineates its posterior border. Consequently, subsequent neurogenesis and cellular behavior in the developing cerebellum is oriented along the rostro-caudal rather than the medio-lateral body axis.

Importantly, the ventricular proliferation region is subdivided by the early genetic patterning events described above into two germinal zones: the ventricular zone (VZ) positioned in the ventral alar plate and the upper rhombic lip (URL) in the dorsal alar plate region (Fig. 2b). Both of these zones continue caudally and can be found in all rhombomeres, yet posterior to the cerebellar primordium, the dorsal zone is termed the lower rhombic lip (LRL). Although these proliferation zones cannot be distinguished visually, they are defined molecularly by the expression of two basic helix loop helix (bHLH) transcription factors. In zebrafish, like in all other vertebrates, expression of the *pancreas transcription factor 1a* (*ptf1a*) gene defines the ventricular zone [15], while expression of *atonal homolog 1* (*atoh1*) delineates the rhombic lip [16, 17], and expression of these transcription factors is juxtaposed but mutually exclusive (Fig. 2c) [18]. Because of genome duplication events in teleosts, zebrafish contains three *atoh1* paralogous genes with partially overlapping but also distinct spatio-temporal expression patterns [19–22].

### Ventricular zone derived cerebellar neurons

Proliferation of neural cells in the VZ is regulated by hepatocyte growth factor (HGF) through Krüppel-like factor 8 (Klf8)-regulated expression of its receptor Met [23, 24]. At 48hpf, *ptf1a* expression in the VZ is prominent in neuronal progenitors that emanate from the ventral alar plate and soon delaminate from the neurogenic zone to migrate dorsally over short distances, during which they become postmitotic [15, 22, 25]. *ptf1a*-expressing progenitors differentiate into the inhibitory neurons of the cerebellum, all of which use gamma-aminobutyric acid (GABA) as neurotransmitter namely Purkinje cells (PCs), stellate cells (StCs), and Golgi cells (GoCs) [25]. Cellular equivalents of basket cells (BCs) have not been identified in the teleostian cerebellum so far and may not exist. Similarly, the existence of inhibitory VZ-derived Lugaro cells and recently characterized inhibitory candelabrum cells of unclear origin has not been described in teleosts so far [26–28].

**Fig. 4:**
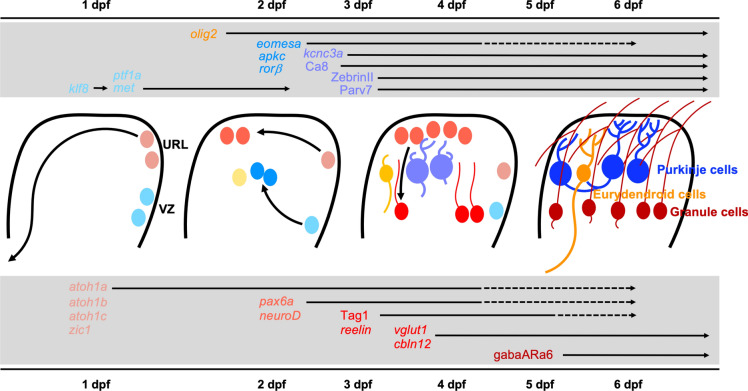
Schematic drawing of developmental steps of cerebellar key neurons and associated gene expression. Granule cells (GCs) as predominant cerebellar interneurons are marked in maroon, Purkinje cells (PCs) as primary output neurons are marked in blue, and eurydendroid cells (ECs) as cerebellar efferent neurons are marked in orange. Sagittal views (anterior to the left) of the developing cerebellum are portrayed with black arrows indicating migration of neuronal progenitors. In the grey box above, expression of key genes in successive stages of PC and EC development is shown, similarly the grey box below lists crucial genes expressed during the developmental course of GCs. Only a selection of commonly used marker genes is shown; for most of these, the exact time of onset and duration of expression have not been systematically revealed. Therefore, a tentative expression onset associated to maturation stages is shown. Solid arrows indicate that expression lasts to adult stages, while arrows with dashed arrowheads indicate that expression in adult stages has not been investigated. *URL* upper rhombic lip, *VZ* ventricular zone

Eurydendroid cells (ECs), the equivalent of deep cerebellar nuclei neurons in mammals, are also mostly derived from the *ptf1a*-expressing VZ, while a minor contribution from the URL to the EC population has been suggested as well [22]. These neurons arise at around 36hpf in the zebrafish cerebellar VZ and can be identified by their expression of *oligodendrocyte transcription factor 2* (*olig2*), which distinguishes them from PCs (Fig. 4). The size of this cell lineage is induced by dorsally derived Wnt1 and suppressed by ventrally derived Hedgehog (Hh) signal transduction events [29]. EC neurons form an exception, as they differentiate into excitatory glutamatergic efferent neurons of the cerebellar cortex despite their initial expression of *ptf1a* [22, 25]. Progenitors of oligodendrocytes share this *olig2*-expressing lineage, but they are few in number and consequently most neuronal populations in the zebrafish cerebellum contain non-myelinated axons including PCs and GoCs [22].

From 3dpf onwards, PCs together with ECs, oligodendrocytes, and astrocytic Bergman glia start to form a continuous layer of cells of roughly one cell thickness across the dorsal region of the differentiating cerebellum, with ECs being positioned ventrally at the border of this layer and reaching occasionally into the granule cell layer (GCL). To this medial layer, PCs are continuously added until about 6dpf, when a plateau with about 400 PCs is reached and the cerebellum matures [30, 31]. Only after PC progenitors have reached the PC layer, they start to differentiate by forming a large dendritic tree and a short unmyelinated axon. Initially, immature PCs form several dendritic neurites until a single primary dendrite is selected mediated by cell autonomous functions of atypical protein kinase C (aPKC) involved in juxtaposing the Golgi apparatus near the preferred dendrite [32]. At this time, PCs express several genes characteristic for this principal neuron of the cerebellum including *eomesodermin homolog a* (*eomesa*), *RAR-related orphan receptor beta* (RORβ), *carbonic anhydrase 8* (ca8), voltage-gated potassium channel protein Kcnc3a, parvalbumin7 (parv7) [31, 33, 34]. Yet, of these, only *ca8* has been functionally investigated suggesting that it mediates survival of differentiating PCs [35]. Mature PCs in zebrafish can be identified immunohistochemically with antibodies against Ca8, Parv7 or ZebrinII (Fig. 4). The latter does not identify subsets of PCs organized in stripes like in mammals, but instead is found throughout the entire PC population [36]. Also, other genes expressed in subsets of zebrafish PCs have not been explicitly identified so far, yet recently four different subtypes of PCs have been revealed in the corpus cerebelli based on the size of their soma, the morphology of their dendritic tree, and their different physiological properties [37]. In the valvula, three different PC subtypes were identified with different properties than PCs in the corpus cerebelli [38]. Identifying molecular differences between these subtypes will be an exciting research activity in cerebellar research.

### Upper rhombic lip-derived cerebellar neurons

Neuronal progenitors emanating from the URL express paralogs of *zic1* and *atonal homolog 1* (*atoh1*) *atoh1a*, *atoh1b* or *atoh1c* in a partially overlapping manner starting around 18hpf [17, 21, 22]. While continuously producing progenitors, the URL generates different neuronal populations over time. Proliferation control of neuronal progenitors in the URL is poorly studied, but investigations on the LRL suggest that Notch signaling maintains rhombic lip cells in a progenitor state [39]. As inhibition of Notch signaling leads to an expansion of early *atoh1c* expression in the URL, these signaling mechanisms seem to be conserved in the cerebellar primordium [21]. In a first sequence, progenitors expressing *wnt1* and *atoh1a* emigrate from the URL toward the MHB where they turn ventrally to leave the cerebellum and to populate the ventral hindbrain tegmentum [22, 40]. Here, they differentiate into glutamatergic and cholinergic neurons of the secondary gustatory/viscerosensory nucleus, the nucleus isthmi, and the superior reticular nucleus, homologous structures of the parabrachial, parabigeminal, and laterodorsal-pedunculopontine tegmental hindbrain nuclei in mammals [40]. Similarly, neuronal progenitors expressing *atoh1c* around 24hpf leave the cerebellar primordium and differentiate into neurons of unclear identity near the locus coeruleus in the tegmental hindbrain. Yet, the early expression domain of *atoh1c* is not confined to the URL but located near the isthmus suggesting a function in arousal pathways [21]. Thus, non-overlapping expression domains of *atoh1* paralogs give rise to extracerebellar structures and form tegmental neurons of different fate.

In vivo time-lapse imaging of *atoh1a*-expressing neuronal progenitors in combination with pharmacological and genetic experiments has provided some insights into the cell biological mechanisms underlying this early URL-derived neuronal migration. The polysialylation of the neural cell adhesion molecule (NCAM) seems to be required for advancing migration, as removal of this posttranslational modification results in cease of migration close to the URL [41]. The speed of migration instead is regulated by Ca^2+^ transients triggered by a pattern of neurotransmitter activity across the cerebellar primordium, which either promote or slow down migration with acetylcholine and glycine exerting a prominent role [42, 43]. Interestingly, the Ca^2+^ transients appear to act on microtubule trafficking to regulate intracellular cargo distribution for controlling migration [44].

Starting at around 48hpf, the URL continues to release migratory neuronal progenitors, and their exit from proliferation is now mediated by craniofacial development protein 1 (Cfdp1) [45]. When granule cells begin to delaminate from the URL, they initiate expression of the proneural bHLH transcription factor *neuroD1* that is often used to identify the granule cell lineage of the cerebellum [18, 22]. These neuronal progenitors move between the URL and the MHB and still proliferate until they reach the MHB within a distance of one or two cell diameters [46]. Here they follow a similar migratory pathway then before, but now these cells remain in the cerebellum and give rise to glutamatergic granule neurons. At this developmental stage, the URL is spatially patterned with progenitors derived from the medial URL populating the corpus cerebelli and laterally derived progenitors forming the granule cell population of the eminentia granularis. The latter together with some cells remaining in the URL to form the granule cell population of the lobus caudalis project to the crista cerebellaris in an ipsilateral and contralateral manner [18]. Studies using transgenic reporter lines confirmed this pattern showing that all three major granule cell populations are formed by *atoh1c*-expressing progenitors [21]. Interestingly, a separate granule cell population expressing *atoh1a* but not *atoh1c* localizes only to the corpus cerebellum, but whether these different granule cell populations serve different functions remains to be clarified. Excitatory unipolar brush cells as URL-derived cerebellar neurons in mice have not been identified and characterized in teleosts so far [26].

Migration of these granule cells occurs glia-independent in contact with each other in chains that are held together by the adhesion molecule Cadherin2 (Cdh2). Besides regulating the coherence of this tangential migration, Cdh2 also controls the directionality of granule cell migration in a cell autonomous manner. Interestingly, Cdh2 is relocated within the membrane of migrating granule cells from the rear to the front during their forward movement likely to align the microtubule cytoskeleton and to reposition the centrosome [47]. When reaching the MHB, differentiating granule cells begin to express the adhesion molecule Tag1 followed by the transcription factor Pax6a, the extracellular matrix molecule Reelin, and the secreted synaptic protein Cerebellin12 [18, 34, 48] (Fig. 2). Reelin has been revealed to position migratory cells derived from the VZ including PCs, ECs and Bergman glia in dorsal positions [49]. The probably most definite evolutionary conserved marker almost exclusively expressed in cerebellar granule cell is the GabaA-receptorα6 subunit, which is maintained by mature granule cells in adult stages [18, 50]. During this first wave of granule cell migration, which lasts until about 6dpf, all granule cell populations are being established and settle in their definite location. Yet, granule cell migration continues during larval stages [18] and is maintained in adult stages from neurogenic regions through the established neuronal layers of the mature cerebellum and is likely to now occur along glial fibers [18, 51, 52].

## Connectivity of zebrafish cerebellar neurons

To elucidate the functions of the zebrafish cerebellum, it is essential to understand the detailed connections of the teleostian cerebellar neurons. In zebrafish, most of the key cerebellar neurons found in mammals have been identified. Also the neuronal morphologies and overall neuroanatomical organization of the zebrafish cerebellum are reminiscent to mammals [4]. This has rendered the easily accessible zebrafish cerebellum as an important model for cerebellar circuit analysis. The zebrafish cerebellar cortex consists of an outermost molecular layer (ML), a medial Purkinje cell layer (PCL), and an internal granule cell layer (GCL) (Fig. 1a). The valvula cerebelli (Va) and the corpus cerebelli (CCe) display these three neuronal layers, whereas the lobus caudalis cerebelli (LCa) and the eminentia granularis (EG) contain only a GCL [3, 25, 53].

### Cerebellar afferents

**Fig. 5:**
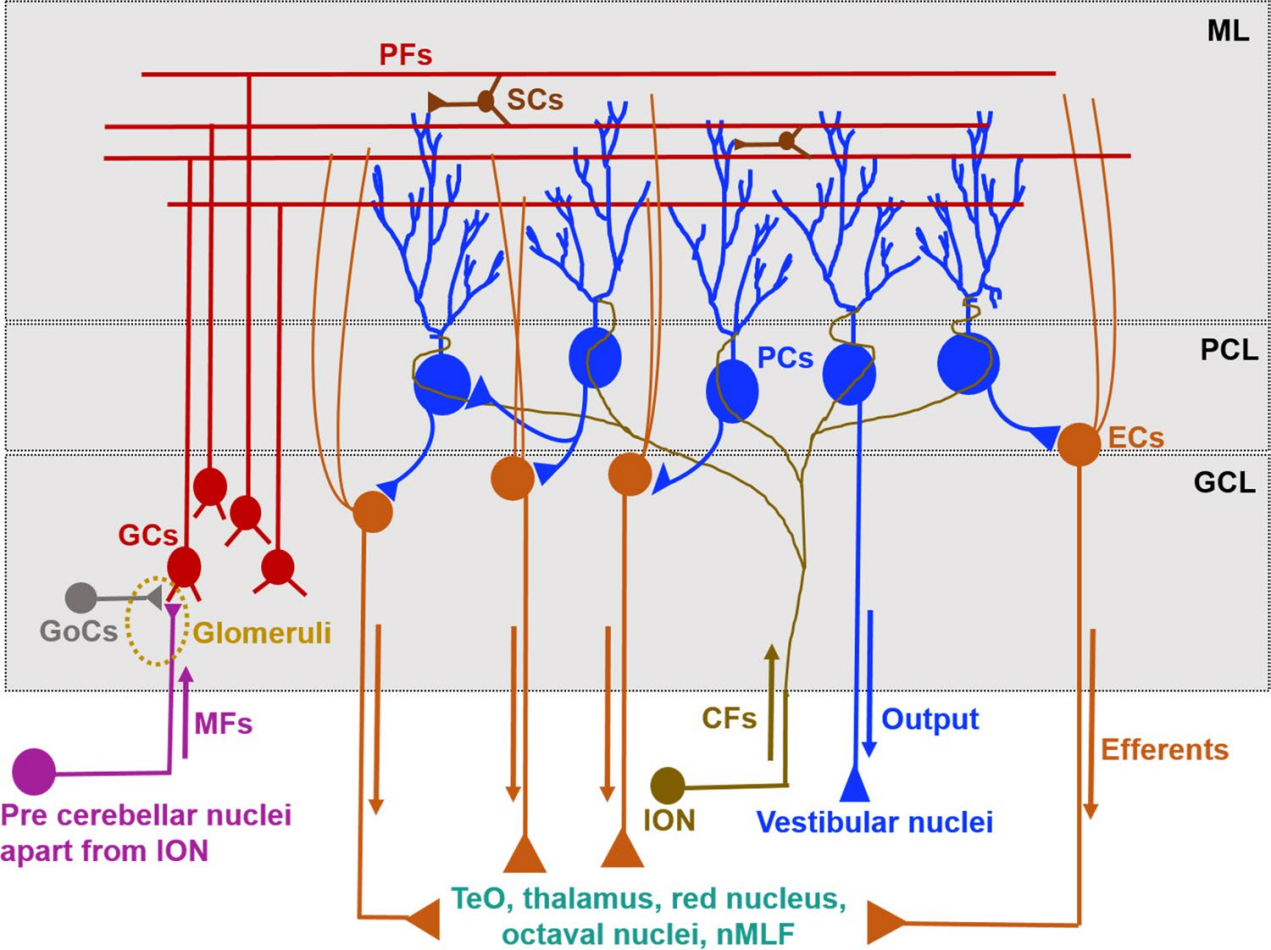
Schematic drawing illustrating intracerebellar connections. *CFs* climbing fibers, *EC* eurydendroid cells, *GCL* granule cell layer, *GCs* granule cells, *GoCs* Golgi cells, *ION* inferior olive nuclei, *MFs* mossy fibers, *ML* molecular layer, *nMLF* nucleus of the medial longitudinal fascicle, *PC* Purkinje cells, *PCL* Purkinje cell layer, *PFs* parallel fibers, *SCs* stellate cells

**Fig. 6:**
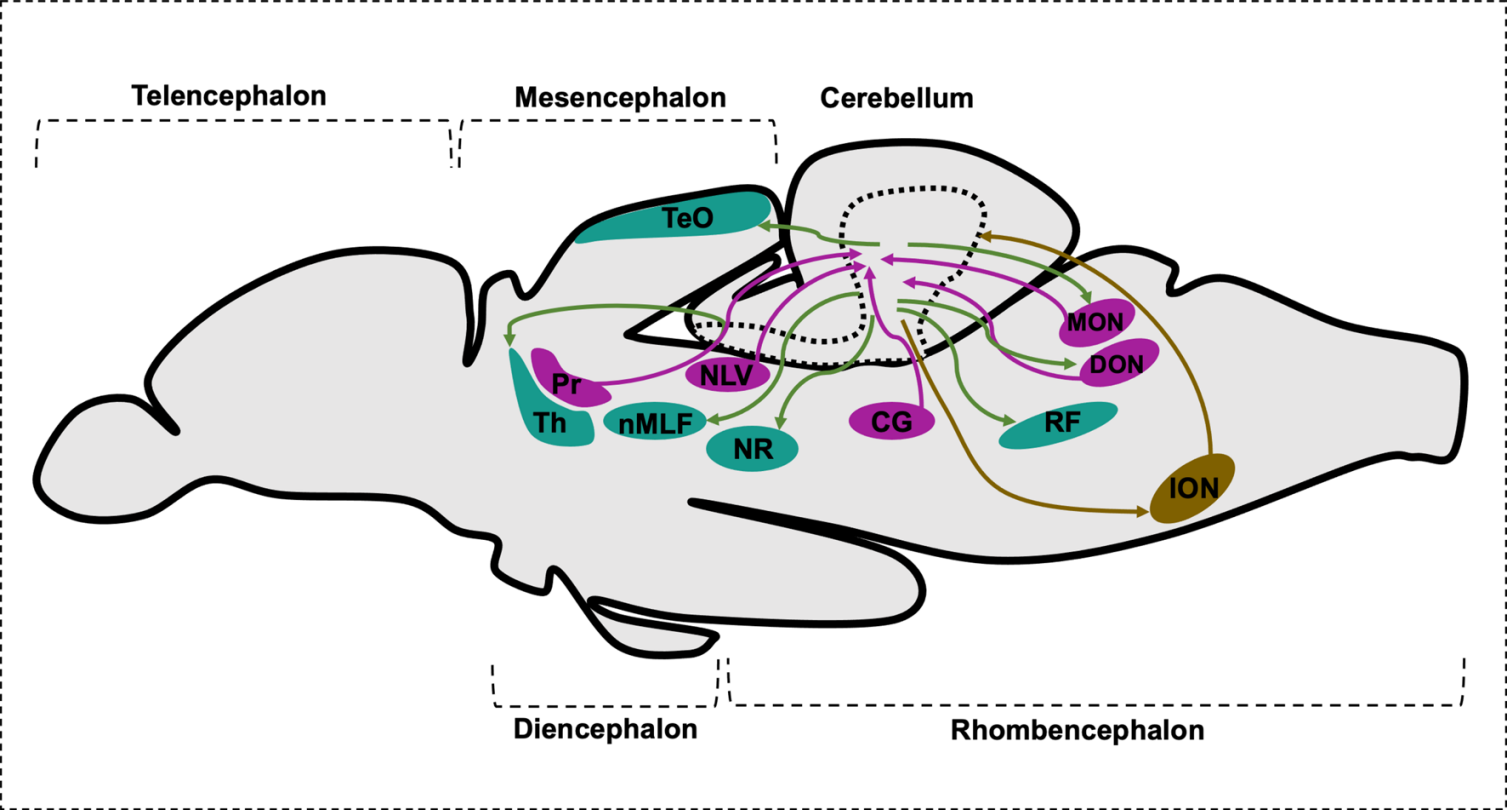
Schematic drawing of sagittal section of brain representing cerebellar afferents and canonical efferent population. *CG* central gray, *DON* descending octaval nucleus, *ION* inferior olive nuclei, *MON* medial octavolateralis nucleus, *NLV* nucleus lateralis valvulae, *nMLF* nucleus of the medial longitudinal fascicle, *NR* red nucleus, *Pr* pretectal nuclei, *RF* reticular formation, *TeO* optic tectum, *Th* thalamus

The cerebellar afferents provide the cerebellar neurons with input from neurons outside the cerebellum known as precerebellar neurons. The two types of afferent fibers to the cerebellum are the mossy fibers (MFs) and the climbing fibers (CFs) (Fig. 5). The MFs are axonal projections emanating from precerebellar nuclei that are located in the diencephalon (pretectum) and the rhombencephalon (Fig. 6). These nuclei are: the central pretectal nucleus (CPN), the intercalated pretectal nucleus (Pi), the paracommissural nucleus (PCN) in the pretectal region and the nucleus lateralis valvulae (NLV). In the rhombencephalon, the medial octavolateralis nucleus (MON), the descending octaval nucleus (DON) as well as the central gray (CG) extend - as precerebellar neurons - their axons to the cerebellum as MFs [54, 55]. CPN and PCN obtain visual inputs from the retina [54], NLV receives input from the telencephalon and hypothalamus [56], MON and DON obtain lateral line and vestibular sensory information, respectively [57, 58], while CG receives input from the habenulo interpeduncular tract [59]. These signals from MFs are conveyed to the cerebellar granule cells. Interestingly, these projections from other brain regions do not relay their information via nuclei in the pons ventrally located to the cerebellum, since teleosts lack explicit pontine nuclei [60], but project directly to the granule cell dendrites.

Monosynaptic retrograde tracing with recombinant rabies viruses detected the inferior olive nuclei (ION) located in the caudo-ventral hindbrain as cerebellar presynaptic neurons [55]. In addition, the retrograde labeling of ION revealed contralateral projections of CFs to the Purkinje cells in the cerebellum [53]. The ION through these CFs activates appropriate cerebellar circuits for successful adaptation of motor programs by calculating error signals during motor learning [61]. Moreover, ablation of ION neurons resulted in severe morphological changes in olivocerebellar circuits [62].

### Intracerebellar circuits

Intracerebellar circuits are mainly formed by granule cells (GCs), Purkinje cells (PCs), Golgi cells (GoCs), and stellate cells (SCs) (Fig. 5a). The glutamatergic GCs as the targets of the MFs are located in the GCL and form two distinct cerebellar circuits in Va/CCe and in EG/LCa regions. The GCs in Va/CCe extend their axons, called parallel fibers (PFs), from the GCL to the ML and provide excitatory input to PCs and likely ECs, whereas GCs in the EG/LCa not only provide input to PCs and probably ECs, but also extend PFs to a dorso-anterior hindbrain region outside the cerebellum, termed crista cerebellaris (CC), to form synapses with dendrites of crest cells whose cell bodies are located in MON [25, 53, 57]. Therefore, some of these GCs also establish cerebellar efferent projections. In addition, the GCL also contains Golgi cells as GABAergic interneurons whose axons terminate on GC dendrites and form a specialized synaptic structure termed glomerulus along with MFs in the GCL (Fig. 5a). Consequently, these neurons modulate GC activity by providing an inhibitory feedback circuit.

The ML consists of PFs, PC dendrites, EC dendrites, and SCs. The ML is the layer in which one of the most prominent cerebellar synapses is formed, the PF-PC synapse, which provides excitatory input to PCs. In addition, imaging studies suggest that PFs provide input to SCs, which extend axons parallel to the PFs and in mice form inhibitory synapses with PC dendrites to modulate PF input [25]. PFs and SC projections, thus, play a central role in the cerebellar circuitry integration and processing of the input information [25, 53]. While PCs extend their dendritic structures to the ML, the soma is located in the PCL. Here, PCs receive excitatory input from CFs; this CF-PC synapse is the second prominent cerebellar synaptic connection. The PCs extend their short axons to ECs that are located near the PCs [63] and some PCs also form interconnections with other PCs forming PC-PC synapses [25, 37, 53] (Fig. 5a). Of note, PCs in the lateral region of CCe send direct efferents to the vestibular nuclei (cerebello-vestibular tract), which may play a role in early vestibular processing [58]. Thus, like GCs, there is a small fraction of PCs that does not act in intracerebellar circuitry but represents an efferent cell population.

### The canonical efferent population

As detailed above, some PC and GC subpopulation provide efferent connections to specific structures in the anterior hindbrain. Yet, the main cerebellar efferents are formed by the ECs that are located at the intersection between GCL and PCL. ECs receive input from PCs and probably GCs and project efferents beyond the cerebellum. Studies in which the genetically encoded transsynaptic anterograde tracer wheat germ agglutinin (WGA) was expressed specifically in PCs were able to identify the efferent cerebellar system in zebrafish by anti-WGA immunohistochemistry. The cerebellar efferent structures included the thalamus, the optic tectum, the octaval nuclei such as MON, DON, the reticular formation, the red nucleus, the nucleus of the medial longitudinal fascicle (nMLF), and others [63] (Fig. 6). Comparisons revealed that zebrafish cerebellar efferents show a similar connectivity as found in mammals [64].

In addition, tracing of individual axonal projections of ECs revealed a regionalization of EC efferents. For example, most of the medial ECs of the cerebellum were found to project to the thalamus through tectal neuropil, whereas medio-lateral ECs projected only to the optic tectum, with axon terminals constrained to deep tectal nuclei [65]. Among the medio-lateral tectal ECs, lateral ECs project to the caudal tectum and medial ECs to the rostral tectum. The ECs markers *olig2* and *calbindin2b (calb2b)* in the cerebellum are expressed in specific regions, where medial ECs express *olig2* and lateral ECs express *calb2b* [25, 29]. This might provide molecular cues to discriminate between different thalamical and tectal projecting ECs [65]. By these EC projections, the optic tectum receives input from the cerebellum and sends output from the deep tectal layers to the motor sensory neurons such as the superior raphe nucleus, the hindbrain reticular formation, the medulla oblongata, and other targets in the hindbrain [66, 67].

Projections of ECs from the caudolateral and caudomedial cerebellum were identified to connect to the octaval nuclei in the ventral hindbrain, the projections are mostly ipsilateral and only few transmit information to the contralateral side. The ECs in the rostromedial part of the cerebellum have long ipsilateral and contralateral projections to the nMLF, to the reticular formation and only contralateral projections to the red nucleus in the midbrain tegmentum [63, 68]. However, how the output from ECs integrates with sensory and motor information in other brain regions is not yet known.

## Zebrafish cerebellar circuitry and its evolutionary conservation

Although it may seem a paradox, the cerebellum is curiously one of the brain structures, most conserved and with highest variability throughout evolution. It emerged during the agnathan–gnathostome transition; as it is present in all jawed vertebrates, from the phylogenetically oldest or most ancient (cartilaginous fishes, including sharks, skates and rays) to the most recent (mammalians and birds) living animals with an actual layered cerebellum [69–71]. All of them share a fundamental cerebellar structure and circuitry, but differences specific for each taxonomic group can also be recognized. Thereby, the morphology of the cerebellum is highly variable, from a highly folded structure in mammals and birds, to a very simple and flat cerebellar cortex in zebrafish [72].

### Conservation and evolution of cerebellar cell types

Despite the high degree of evolutionary conservation with respect to a layered structure, the distribution and shape into three layers (molecular, Purkinje, and granule cell layer) differ among species. The distribution from external or marginal to internal side of the cerebellar cortex in the zebrafish is akin to that in mammals with granule cells underneath the PC layer, but a clear white matter area as in mammals is not present in teleosts [72]. On the other hand, more evolutionary ancient fish present special features, such as basal bony fishes with PCs not aligned in a layer but distributed in clusters [73], besides in cartilaginous fish in which the granular layer is grouped into two paramedian eminences and the PC layer is laterally located, these cartilaginous fish show a much wider ventricular space than bony fish. Another curious layering distribution is found, for example, in reptiles with an everted cerebellum, and presenting the granule cell layer toward the marginal side [72].

**Fig. 7:**
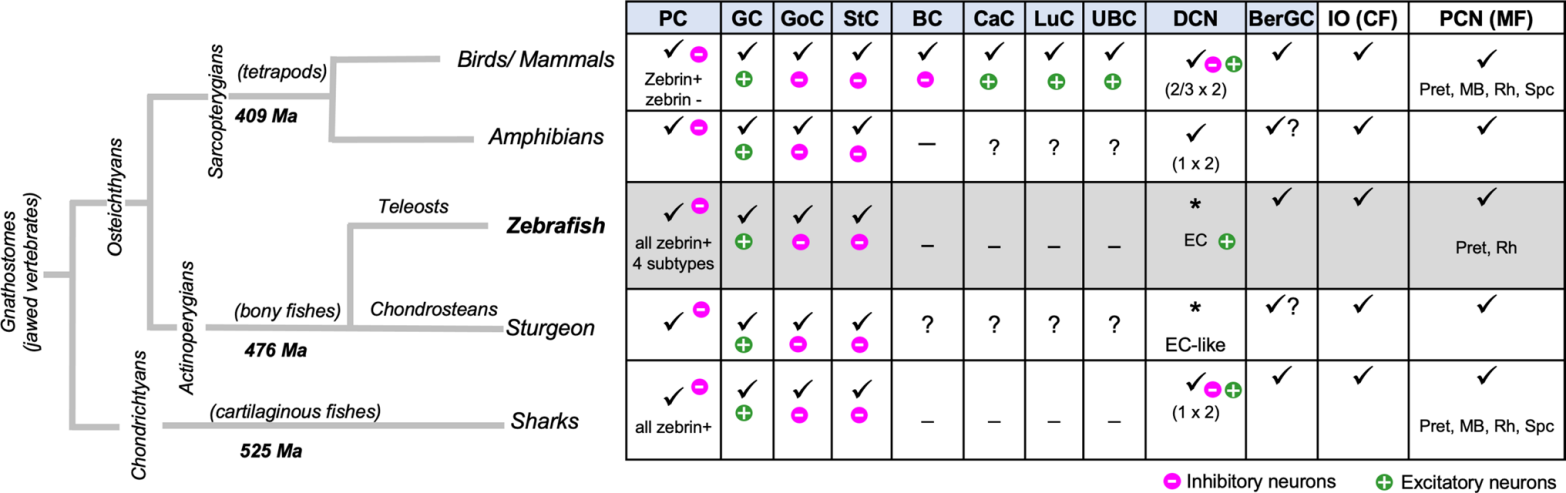
Evolutionary conservation of the main cell types in the cerebellar cortex comparing the zebrafish with those in other groups of jawed vertebrates. *BC* basket cells, *BerGC* Bergmann glial cells, *CaC* candelabrum cells, Cb cerebellum, *CF* climbing fibers, *DCN* deep cerebellar nuclei, *EC* eurydendroid cells (*equivalency to DCN), *GC* granule cells, *IO* inferior olive, *LuC* Lugaro cells, *Ma* million years ago (molecular dating for nodes of divergence), *MB* midbrain, *MF* mossy fibers, *PC* Purkinje cells, *PCN* precerebellar nuclei, *Pret* pretectum, *Rh* rhombencephalon, *Spc* spinal cord, *StC* stellate cells, *UBC* unipolar brush cells. Information based on: [72, 83–85]. Additional literature was also taken into consideration (see text)

The Purkinje cells (PCs) in zebrafish are considered, as in all groups of gnathostomes, the main cerebellar neurons and represent the major processing information center of the cerebellum. However, this neuronal population, instead of displaying a compartmentalization of a zebra-like pattern as in mammalians (due to two types of PCs, Zebrin-II-positive and Zebrin-II-negative), all Purkinje cells (PCs) are Zebrin-II (or Aldolase-C) immunoreactive (Fig. 7; [36]), as it is the case for cartilaginous fish [74, 75]. Yet exceptions exist in certain bony fish, where some PCs were reported as Zebrin-II-negative, but the PC population is still devoid of a banding pattern compartmentalization [76]. Notwithstanding, independently of Zebrin-II expression, there is evidence that the PCs in zebrafish do not form a homogeneous cell population. Indeed, recent studies in the mature cerebellum reported PC subtypes involved in locomotor and non-locomotor behavior, respectively [37, 38].

Efferences of PCs in teleosts are unique. Instead of being organized in nuclei (such as the deep cerebellar nuclei or DCN in amniotes, containing excitatory and inhibitory neurons), the zebrafish has eurydendroid cells, a scattered cell population spread across the cerebellar cortex, laying in the vicinity of PCs. These particular PC efferences in teleost fishes are considered homologous to the DCN of amniotes, since they implement the same main functions, receiving the output from PCs and sending connections to extracerebellar structures. Certainly, they could correspond to a modified version by evolutionary divergence from the original or ancestral cerebellar nucleus. Indeed, going back in the evolutionary history, cartilaginous fishes (the most ancient extant organisms with cerebellum) show a well-defined cerebellar nucleus with subdivisions [77, 78]. Then the cerebellum of sturgeon (which belongs to the most ancient radiation of bony fishes) contains eurydendroid-like cells, grouped in three regions of the corpus cerebelli, and with less branching than in zebrafish [79]. Furthermore, the ECs in the basal actinopterygian fish *Polypterus senegalus* present some similarities to teleosts as well as DCNs in mammalians [73]. Thus, this cell type in the most ancient bony fish weas proposed to correspond to an intermediate step before the emergence of the actual eurydendroid cells in teleosts [73].

Regarding other cerebellar cell types, there are some interneurons in the amniote cerebellum that are generally lacking in fish (Fig. 7). Basket cells, present in mammalians, are absent in bony and cartilaginous fishes. Albeit a specialized subgroup of stellate cells, such as the deep stellate cells in the valvula of the cerebellum in mormyrids could serve this function [80]. Extra glutamatergic interneurons also form part of the cerebellum in mammals, including unipolar brush cells, Lugaro cells, and candelabrum cells. In fish, these cell types were not reported, with the exception of the mormyrid electric bony fish [80], and an Opisthocentrus—*Pholidapus dybowskii—*[72], in which unipolar brush cells were detected; as well as Lugaro cells in the teleost *Pholidapus dibowskii* [81].

The cerebellar glial cells are also quite evolutionary conserved, as the Bergmann glia (the cerebellar radial glia) are present in zebrafish like in the cerebellum of other vertebrate groups, and express specific markers, such as the brain lipid-binding protein (BLBP) or fatty acid binding protein 7 (FABP7), and glial fibrillary acidic protein (GFAP) [25]. Other glial cell types include oligodendrocytes and astrocytes. Although zebrafish does not show cells with typical astrocyte-like morphology, it has been recently reported that, indeed radial glial cells of the zebrafish brain could carry out functions equivalent to astrocytes of the mammalian brain [82].

### Intracerebellar and extracerebellar connectivity in zebrafish, an evolutionary perspective

**Fig. 8:**
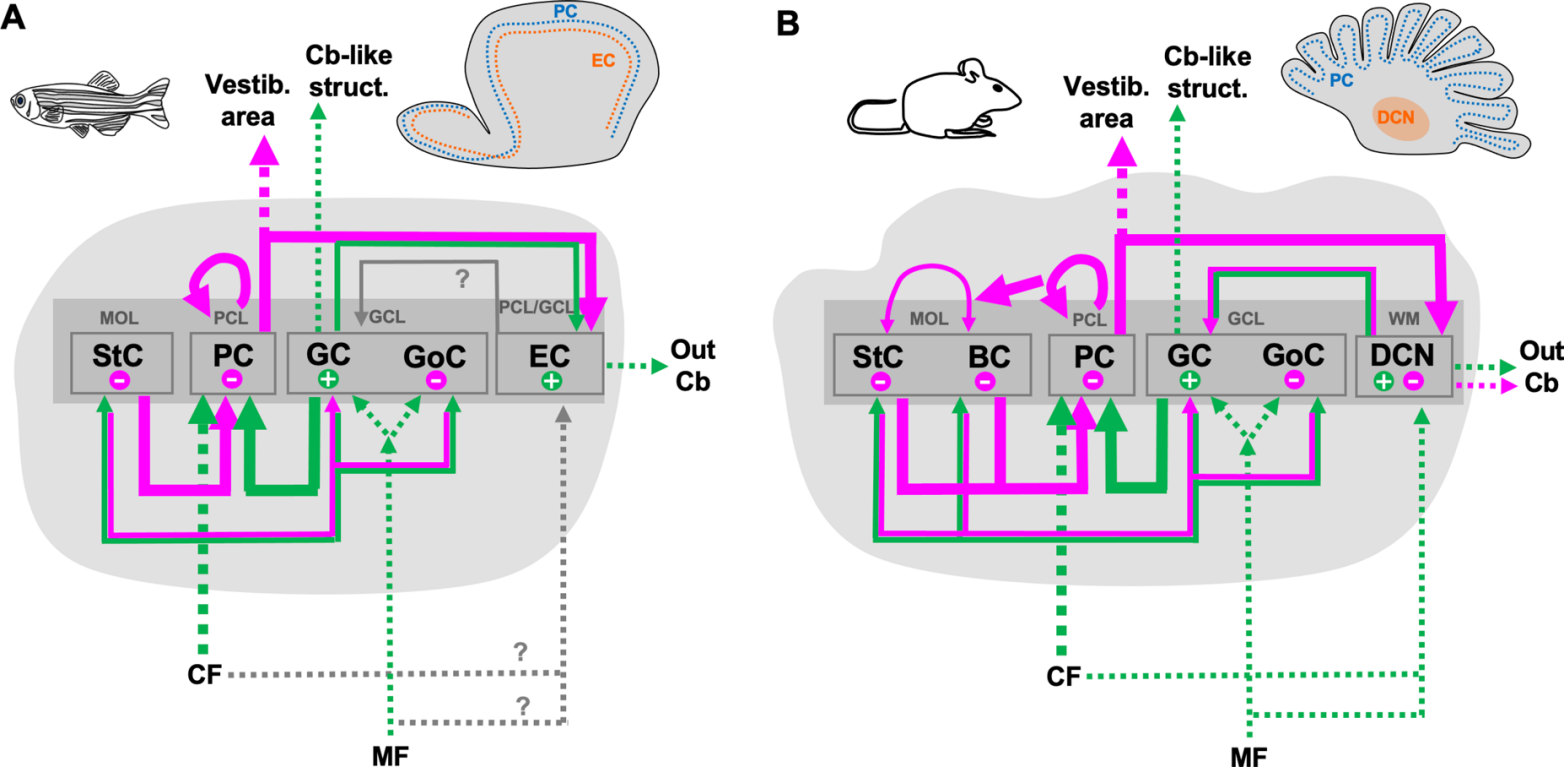
Evolutionary conservation of the cerebellar circuitry in zebrafish. Schematic drawing of the circuitry in the cerebellum of zebrafish (**a**) compared to that in mouse (**b**). Thick lines indicate direct connections of the Purkinje cells. Green arrows indicate intracerebellar excitatory input connections, while magenta arrows symbolize intracerebellar inhibitory input. Extracerebellar input or output instead is shown by dashed arrows with excitatory connections shown in green and inhibitory ones marked in magenta. *BC* basket cells, *Cb* cerebellum, *CF* climbing fibers, *DCN* deep cerebellar nuclei, *EC* eurydendroid cells, *GC* granule cells, *GCL* granule cell layer, *GoC* Golgi cells, *MF* mossy fibers, *MOL* molecular layer, *PC* Purkinje cells, *PCL* Purkinje cell layer, *PCN* precerebellar nuclei, StC stellate cells, *WT* withe matter. Information mostly based on: [72, 83]. Additional literature was also taken into consideration (see text)

The basic circuitry of cerebellar cells is also highly preserved throughout evolution. In all groups of animals, including zebrafish, the cerebellar information is mostly processed by the PCs, the main neurons that receive directly or indirectly input from intracerebellar interneurons, and from extracerebellar or precerebellar nuclei (Fig. 8).

The intracerebellar connections within the zebrafish cerebellar cortex are akin that in other groups, as for PCs receiving their main input from granule and stellate cells, and sending their output to the efferent cells, with the exception of a group of PCs located in the corpus cerebelli that directly project to the vestibular nuclei [86]. Interestingly, PCs are also interconnected with other PCs by axonal collaterals, which allows to synchronize the cerebellar network (Fig. 8). This is the case for all the PCs subtypes of the corpus cerebelli described in the mature cerebellum [37, 38]. Collaterals of PC axons in mice interconnect with other PCs and interneurons as well [87]. As for the most abundant interneurons, the granule cells, they distribute the received information through their axons (parallel fibers), not only to the main neurons—the PCs—but also to other cerebellar interneurons (stellate and Golgi cells). Additional direct output through the parallel fibers from the caudolateral lobe is sent out to cerebellar-like structures, i.e., to the crest cells in the dorsal hindbrain (reviewed by [25, 70]). Akin projections are also present in cartilaginous fishes [88]. Furthermore, recent physiological studies suggested a direct output from granule cells to eurydendroid cells [89], but neuroanatomical evidence for GC-EC synapses is still missing. In other groups of animals, direct connections between the granule cell layer and DCN were confirmed as well, as those found in mammalians, but these consist of nucleo-cortical projections (from the DCN to the cerebellar cortex), ending as mossy fiber afferents [90, 91]. Nucleo-cortical connections were also detected in cartilaginous fish [92, 93].

With respect to the extracerebellar input information, the two main types of afferents (mossy and climbing fibers) in zebrafish are comparable to all groups of animals with a cerebellum (Fig. 8). A third type of cerebellar input is represented by the neuromodulatory fibers, ending in the three layers of the cerebellar cortex, but which appeared less common and were reported only in some species (reviewed by [94]); e.g., projections from the locus coeruleus, displaying multi-layered ending in the cerebellar cortex [72]. Likewise, catecholamine fibers from the locus coeruleus were detected in the Purkinje and granule cell layers of the zebrafish cerebellum, where a strong density of noradrenergic receptors is present [95, 96].

The majority of afferent inputs ends on the granule cells as mossy fibers; and whose information is then indirectly transmitted to PCs. Even though, most of the precerebellar nuclei (origin of the mossy fibers) correspond to those in other cerebellated animals, two additional precerebellar nuclei present in zebrafish but not in other groups are: the nucleus valvula lateralis and the nucleus paracommissuralis. Moreover, the exceptional large size of the cerebellum in some animals implies further cerebellar connections, such as the pontine nuclei in mammals and birds, due to the lateral expansion of the cerebellum in these animals [72].

Regarding the well-known direct connection between climbing fibers and PCs, while in mammalians, the climbing fibers reach distal branches of the PCs dendritic tree, in teleosts, they extend only until the basal dendrites. On the other hand, in basal bony fishes and in cartilaginous fishes, climbing fibers only reach the soma of PCs, reminiscent of the early developmental stages of climbing fibers in mammalians [73]. Whether a topographic map projection between cell subpopulations of inferior olive and cerebellum occurs in zebrafish like in mammals is not known yet. Nevertheless, physiological studies in the zebrafish larvae revealed regionally distinguishable sub-populations of PCs [63, 97, 98], as well as granule cell clusters in the different parts of the cerebellum [99]. This could suggest distinctive efferent projections, as well as afferent connections (via climbing and/or mossy fibers) ending on specific areas of the corpus cerebelli in the zebrafish.

Overall, the zebrafish cerebellum shows some particularities specific of ray-finned fishes (e.g., unique PC efferences), likely due to a partial evolutionary divergence or secondarily derived features in this group. However, most of its cerebellar scaffold, such as main cell types and their connectivity, is highly conserved compared to that in other vertebrate groups, thus prone to form part of the fundamental network of the vertebrate cerebellum. Because of the numerous genetic tools and state of the art techniques available in zebrafish, the research on the cerebellum of this model organism is valuable for addressing functional studies, to obtain a deeper understanding of the intricate operational or physiological mechanisms of the circuitry in the cerebellar system.

## Physiology and function of cerebellar Purkinje cells

The zebrafish cerebellum is considered to regulate body posture, to coordinate directed movements, to mediate motor learning, and to serve higher socio-emotional functions such as exploration or anxiety, analogous to conserved cerebellar functions in other vertebrates. The conserved circuitry is well designed for exerting these functions. Therefore, on the electrophysiological level, a well-functioning, fine-tuned but still highly plastic cerebellum is essential for the zebrafish, because sensorimotor behaviors need to be permanently adjusted during development, experience, environmental changes, social interactivity, and learning so that the organism can optimally adapt to changes from outside as well as self-induced changes [61, 63, 97, 100–102]. In a developmental context, the cerebellum may be actively engaged in refining and maintaining sensorimotor behaviors as the physiology of neural circuits, muscles, and sensory appendages mature [97]. For example, larval zebrafish increase in length by 60% from 15 to 30 dpf and undergo dramatic changes in tail, anal, and dorsal fin development over the same time period [103]. Therefore, an important role for the differentiating cerebellum in adapting motor output would seem to be particularly important for the zebrafish larva [104].

The main responsible neurons, which are required for the correct processing of neuronal activity within the cerebellum, are the highly conserved Purkinje cells [33, 105]. These cells are also by far the most studied and best understood neuronal cell type in the zebrafish cerebellum regarding their electrophysiological properties [97, 99, 101, 104, 106, 107]. As for other zebrafish cerebellar neurons, almost no electrophysiological data are available, this review will focus on the physiological activity of PCs. The activity of Purkinje cells regulates both practiced and new movements [108, 109]. PCs are influencing motor behavior via two distinct spike forms, which is a characteristic shared by all vertebrates [101, 106, 110–113].

**Fig. 9:**
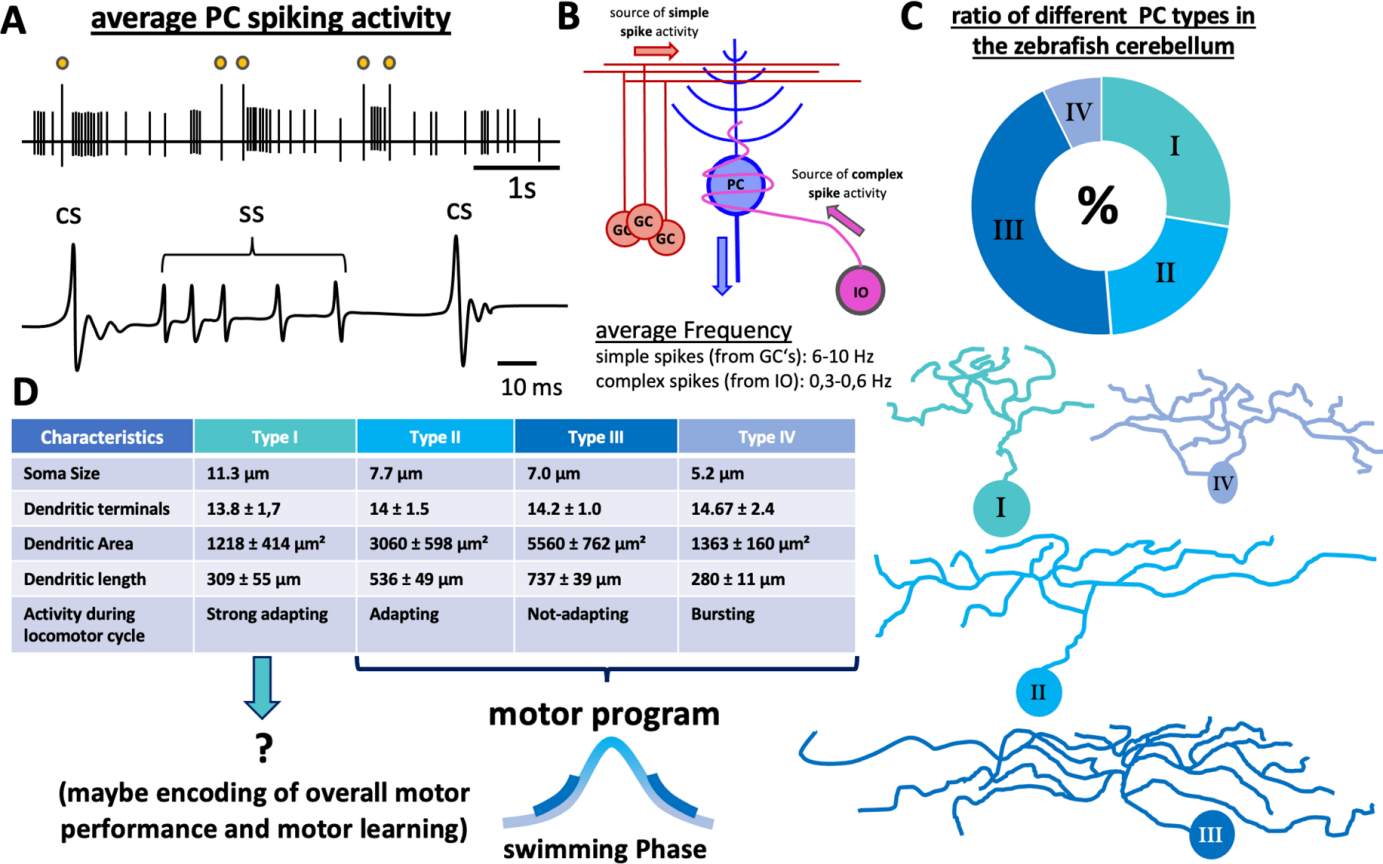
Characterization of Purkinje cell activity and morphology. **a** Schematic drawing of an electrophysiologic recording performed in a healthy PC at 8dpf. The yellow dots mark complex spikes. The trace underneath shows a detailed view of simple spikes and complex spikes. **b** Schematic drawing of simplified PC circuitry, showing the two main input sources—parallel fibers (PF) from granule cells (GCs) and climbing fibers (CF) from the inferior olive (IO). **c** The numerical distribution of the different PC subtypes in the zebrafish corpus cerebellum and schematic drawings of the different morphology of each type. The table in **d** lists other characteristics of the four different PC subtypes. *CS* complex spike, *GC* granule cell, *IO* inferior olive, *PC* Purkinje cell, *SS* simple spike. The graphics and information shown in **c**, **d** were taken and adapted from [37]

The first form is called simple spikes (SS) and they make up the majority of PC activity. Simple spikes are derived by synaptic input from excitatory granule cells at the PF-PC synapses formed at the PC-dendrites (Fig. 9a, b) or can occur spontaneously in a self-evoked manner and may be modulated by stellate cells—inhibitory interneurons [114, 115]. These simple spikes convey primarily motor information which encode mostly velocity, orientation, muscle activity, and direction of the fish. This spike form is mediated by AMPA receptors and is categorized as weak glutamatergic excitation event. Because of the high density of PF-PC synapses, these events occur with a relatively high average tonic frequency of 6–10 Hz in zebrafish larvae and can show bursting activity with frequencies above 50 Hz [97, 101, 104]. In electrophysiological recordings, the waveform of simple spikes is characterized by a single peaking depolarization event followed by a weak hyperpolarization which returns smoothly to the resting potential (Fig. 9a). The resulting activity alters firing patterns of PC efferents (mostly ECs and PCs, despite the direct PC output to the vestibular nuclei), whose output eventually generates mostly tail, fin, and eye movements [97, 101, 116–118].

The second form is called complex spikes (CS) and they arise from synaptic input from climbing fibers (Fig. 9b) and convey mostly sensory information [97]. The CF-PC synapses are also one of the strongest excitatory synapses in the CNS and are formed directly at the soma of PCs [107], therefore having the highest possible impact regarding the changes in membrane potential. The basal firing rate for complex spikes is much lower than the frequency of simple spikes with CS firing on average at rates of 0.3–0.6 Hz in mature PCs of larval zebrafish [97, 101, 104]. When compared to simple spikes from the same recording, complex spikes show nearly double the size in amplitude. The course of a CS is also different and characterized by a huge single peaking depolarization event, which leads to a strong hyperpolarization event with a wavy discharge in both, depolarizing and hyperpolarizing direction until the resting potential is reached again (Fig. 9a). Complex spikes are categorized as strong glutamatergic excitation events and like simple spikes, they are also post-synaptically mediated by α-amino-3-hydroxy-5-methyl-4-isoxazolepropionic acid (AMPA) receptors, which is another electrophysiological feature shared with mammalian PCs [107, 119]. CS travel along the membrane of PCs via voltage-gated Kv3.3 channels, which are highly expressed in PCs and control the complex waveform, which is eponymous for this spike form [120, 121]. Mutations in the coding DNA for Kv3.3 (*KCNC3*) result in a unique neurodegenerative disease termed spinocerebellar ataxia type 13 (SCA13) in mammals (including humans), a disease that can be genetically modeled in zebrafish [121–123]. This suggests that the electrical properties and the expression of specific ion channels which control the activity patterns are highly conserved between zebrafish and mammalian Purkinje cells.

The cerebellar layers and synaptic contacts onto Purkinje neurons are observed as early as 4 dpf [106]. Calcium imaging in 6–7 dpf larvae has revealed that Purkinje neurons are already active during optomotor and optokinetic responses at this developmental timepoint [61, 63]. Electrophysiology recordings in larval zebrafish PCs have shown that multimodal sensory input from PF and CF, as well as the bistable character (which will be explained in more detail below) are already present in 6–8 dpf larvae [101, 104, 106, 107]. Therefore, it takes Purkinje cells in the zebrafish cerebellum only 24–48 h after onset of differentiation to reach maturity and to display spontaneous tonic firing of simple spikes together with more rarely occurring complex spikes [106, 124]. These findings all correlate perfectly with the time when zebrafish larvae are freely swimming and have to begin hunting their prey and avoid predators to survive [125] and demonstrate that the zebrafish cerebellum reaches functional maturity relatively early during development. This rapid time course makes zebrafish advantageous for electrophysiological in vivo studies of cerebellar development and functions in awake animals, which is relatively easy in this model organism and requires only a minimal invasive surgery [97, 99, 101, 106, 126]. In contrast, in rats, cerebellar cortical layers are not evident and Purkinje cells are not functionally mature until ~ 2–3 postnatal weeks, which corresponds to ~ 5–6 weeks post-fertilization [127]. The rapid development of a functional cerebellum is likely to be essential for the survival of the zebrafish larvae, which develops entirely outside of their mother’s body, where they must avoid predators and find food relatively early in life.

The visuomotor processing of PCs might seem abstract, but in simplification, it compares the function in the broadest sense to a parking sensor in a modern car. Both obtain numerous complex information of two main sources but generate only a single and simple form of output. Like mentioned above, the first strong input source is represented by the granule cells (GC) and their axons—the parallel fibers—which are responsible for encoding velocity, orientation and direction of the fish, muscle activity and other motor-related context. The second source of input is the climbing fibers, which originate from the inferior olive (IO) and convey mostly sensory and visual information, which are permanently updating the brain about transient changes in motion, while hunting prey, exploring or escape maneuvers and have, therefore, strong ethological relevance. The same applies to a parking sensor which processes the direction and velocity of the car and the visual input from the camera, which recognizes objects in front of it. Like the parking sensor, healthy PCs also show constant tonic firing activity at a low frequency to show that the “system” is working and if something changes in the visual field, the frequency rises and eventually becomes a permanent high-frequency bursting activity to signal either danger or attention. Therefore, like in a parking sensor, two diverse and complex input sources with many different variables/information are pruned down and therefore simplified by PCs and reduced to a single output signal and therefore makes fast decisions possible, which can decide about life and death of the fish in dangerous situations but also about success when hunting prey or during mating.

**Fig. 10:**
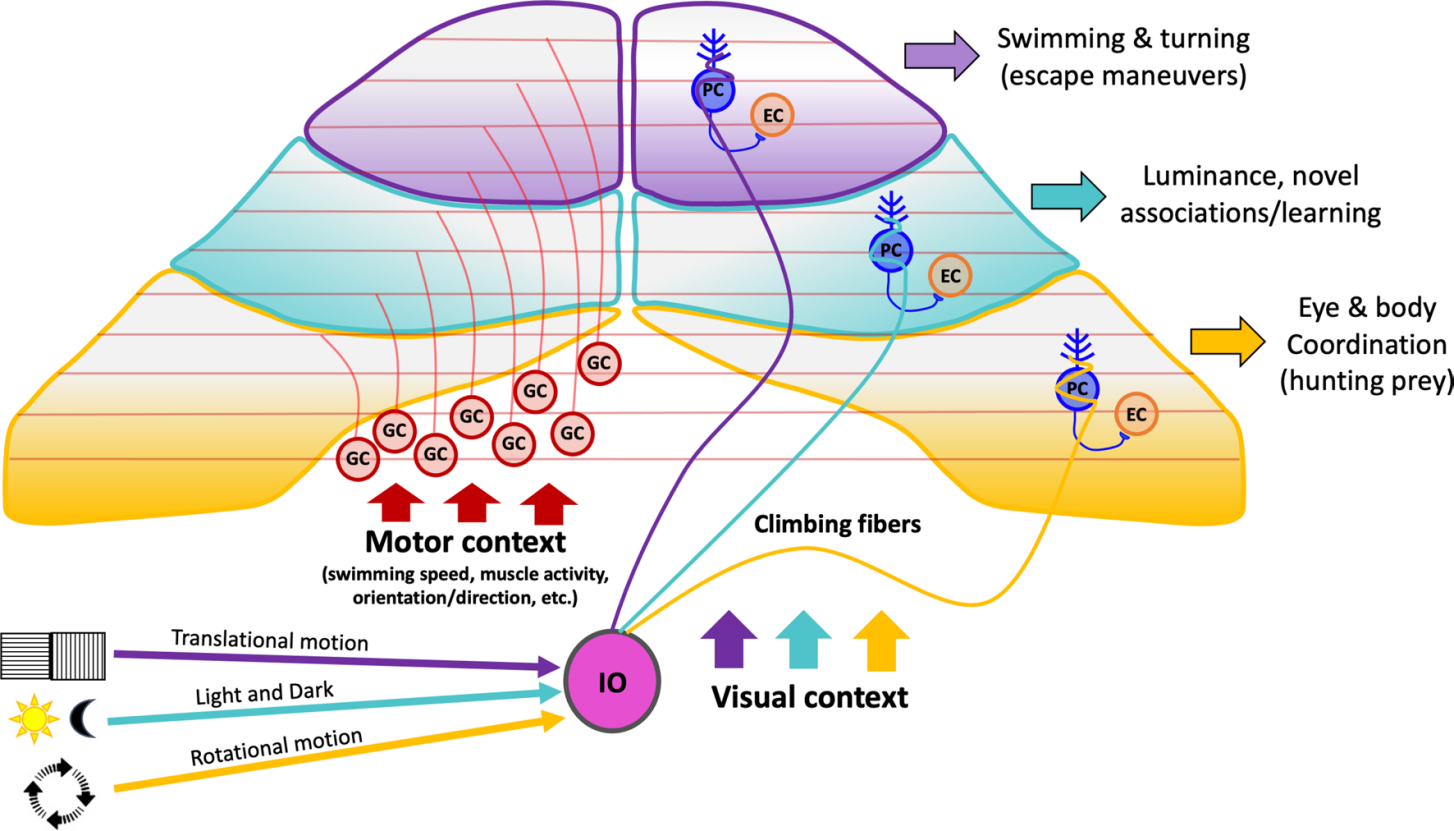
Regionalization of Purkinje cells. Schematic drawing of the region-specific Purkinje cell activity triggered by visual stimuli. PCs in the rostromedially region are primarily active when translational motion is detected by the visuo-sensory system and are involved in overall swimming and turning maneuvers (purple). Purkinje cells in the medial region are mostly active when luminance changes are detected and also during motor learning (cyan). The PCs in the caudal region are most prominently active when stimulated by rotational motion and are mainly involved in precise eye and body coordination (yellow). *EC* eurydendroid cell, *GC* granule cell, *IO* inferior olive, *PC* Purkinje cell. This figure is based on the findings of [97] and was just slightly adapted to fit the style of this review

The localization of a PC is also important for the way how information is processed, because there exists some region and stimulus specific processing, localization and clustering across the cerebellum. This functionally defined regionalization is based on complex spike responses to specific sensory information, affecting the associated motor-related information about body and eye movements transmitted via simple spikes [128]. The sensory information conveyed by CSs are permanently updating the cerebellum about transient changes in motions. Based on the respective stimulus, this visuomotor processing takes place in specific PC layer regions, which likely represent behavior modules which are responsible for sensorimotor integration and motor learning in the cerebellum—another characteristic which they could share with mammals [97, 129]. Differences in developmental timing are known to contribute to the formation of a topographic functional map in the cerebellum across species [63]. Different studies suggest that patterns in the complex spike activity divide the cerebellum spatially and functionally into three regions along the rostrocaudal axis [97, 101] (Fig. 10 adapted from [97]). Complex spikes of Purkinje cells in the rostromedial cerebellum reliably encode acute, directional changes of motion in the visual field with a preferred directional tuning (Fig. 10). This directionally selective motion processing is comparable to the directionally tuned Purkinje cells in the oculomotor vermis of posterior lobes VI and VII in primates, where complex spike activity is able to arrange PCs into functional groups whose simple spikes encode real-time eye motion [97, 130, 131]. Purkinje cells in the central area of the larval zebrafish cerebellum preferentially showed CS activity correlating to conditioned visual stimuli during associative learning. Together with the fact that these PCs are born later in development compared to the other two areas [30], it is speculated that this region may preferentially contribute to flexible or learned sensorimotor behaviors (Fig. 10). PCs in this area also show differences in responses to luminance changes, including light/dark preference, tonic/phasic responses, latency from stimulus onset to complex spike and receptive field size; therefore, they are also thought to process light-mediated behaviors in the larval zebrafish, driving, for example, the circadian rhythm and motivate feeding and exploratory behavior during daytime [97, 132]. In the third area, which is located in the caudolateral region of the cerebellum, PCs respond strongly to unidirectional rotational motion and the axons of these neurons project primarily to the vestibular nuclei [63]. These PCs show tonically elevated CS rates when exposed to visual motion in a temporal to nasal direction presented to the ipsilateral eye (Fig. 10). This region can be seen as homologue to the mammalian flocculonodular lobe, where CS activity conveys information about ongoing, opposing directional visual and rotational head motion, which is used for vestibulo-ocular coordination [97, 133, 134]. Remarkably, these PCs show CS responses not only to rotational but also translational moving fields, which is a characteristic shared with pigeons but not with terrestrial mammals [135]. This different processing can be traced back to the additional complexity of optic flow which comes along with the advanced navigation in the three-dimensional world of birds and fish, which are not bound to the ground like most mammals and can also move up and down in the *Z*-direction.

Even though a lot of information about PCs in the zebrafish cerebellum were gathered over the last decades, they are still up for new surprising discoveries. It was commonly believed that zebrafish PCs are a homologous group of neurons, which all share the same morphology regarding their soma size and dendritic arbors, and therefore also information processing was believed to occur in all PCs in a similar way. But recently it was shown that this is not the case and that even though all PCs in the zebrafish are Zebrin-II positive and indistinguishable by genetic markers known so far [2, 36], they still show strong differences in morphology and activity patterns, so that the PCs in the adult zebrafish cerebellum can be divided into four subtypes [37]. While type I and IV show rather confined dendritic arbors, the dendritic trees of type II and III extend very far and cover large areas in the adult cerebellum (Fig. 9c, d). These different morphological profiles and soma sizes also lead to distinct firing patterns and information processing. Type I and II are considered as (strong) adapting types, because they discharge action potentials with elevated spike frequency during movements. Type III can be seen as not-adapting, because they fire tonically and not elevated during movement episodes. PCs of type IV display a pronounced high-frequency bursting activity. This leads to the assumption that the different types probably also perform specialized functions during the locomotor cycle (Fig. 9d). Yet, even though the PC population seems to be organized into physiologically distinct subtypes, these types are not topographically clustering to create functional areas of the same type but are rather randomly distributed regarding their numbers, ratio, and also their location over the PC layer of the corpus cerebelli [37]. These four subtypes were discovered in adult zebrafish, and these different subtypes are likely to already exist in the larval brain [113] before the cerebellum grows drastically in size and changes its shape.

Another electrophysiological characteristic shared between mammalian and zebrafish PCs is that both show functionally relevant bistability, that is driven by olivary input (= climbing fibers/complex spikes). Bistability means that these neurons can be found either in the tonic or bursting mode (Fig. 9a), and they have the ability to switch from one mode to the other either spontaneously or by strong AMPA-receptor-mediated excitation from climbing fibers which is sufficient to trigger bursts [97, 107, 136, 137]. It is hypothesized that mode switching could serve as a ‘clutch’ that engages or disengages specific Purkinje neurons located in specific regions or modules to control and process locomotor behavior. By switching mode from tonic to bursting, Purkinje neurons might choose to “listen in” on locomotion-related neuronal inputs and get active when stimuli come together which are specific for single PCs or even PC clusters [107].

But vice versa, it is also possible for simple spikes to actively change the impact of complex spikes during elevated motor-activity periods. Like mentioned above, during non-locomotor periods, CS activity can consistently increase or pause simple spiking for several hundreds of milliseconds in PCs. However, in PCs which fire with high simple spike rates during a motor-related task, a CS is only able to reset SS activity for a short time window (< 50 ms) before simple spikes return to their previous high frequency. The narrowing of this temporal window may serve to make finer adjustments of motor activity through very acute perturbations in network activity [97]. Bistability may also have a key role in the short-term processing and storage of sensory information in the cerebellar cortex [138]. In zebrafish, GABAergic inhibition or NMDA-receptor-dependent excitation (key factor for LTP/LTD) seem not to be crucial for generating bursts in PCs [138]. But it was shown that communication between Purkinje cells depends mainly on inhibitory GABAergic transmission and also by direct contact via gap junctions, which form electrical synapses [37, 107]. This mutual inhibition driven by interconnectivity of PCs of the same area is essential for coordinated synchronous activity [37]. So, when performing patch-clamp recordings in a healthy PC, one should expect to see a firing pattern that changes between a rather low amount of actions potentials (= tonic phase) and extremely high frequent activity (= bursting phase) (Fig. 9a).

Currently, although much electrophysiological information was gathered for PCs, other cerebellar neurons of zebrafish are by far understudied. Yet their ease of access should provide valuable insight into the function of cerebellar circuitry in the future and represents a rewarding field of study, because in zebrafish molecular analysis, physiological and behavioral function can be easily combined for establishing true molecule to physiology and function relationships directly in vivo.

## Outlook

Compared to cerebellar research in rodents and birds, investigations on the zebrafish cerebellum are still immature and knowledge about teleostian cerebellar development, physiology, and function is fragmented. Yet, the past years of zebrafish cerebellar research have laid a solid foundation for further studies. Moreover, the powerful combination of molecular manipulation with high-resolution in vivo imaging, electrophysiology and behavioral analysis possible in this model allows for contributing significant and unique insights into cerebellar properties and functions. Of note, the contributions of the cerebellum to vertebrate brain function and information processing are far from being understood and the traditional view about the cerebellum mainly controlling the smooth execution of movements is under revision since some time, ascribing the cerebellum a much larger portfolio of responsibilities in the central nervous system than previously thought. Studies on the zebrafish cerebellum could not only complement understanding of cerebellar development and function but also serve to model cerebellar diseases, to elucidate disease-causing cell biological mechanisms, and provide genetic in vivo models for developing therapeutic strategies by means of genetic interference or pharmacological compound testing and validation. Therefore, cerebellar research in zebrafish is a promising research field for new generations of neurobiologists.

## Data Availability

Not applicable.
